# Joint analysis of multiple phenotypes: summary of results and discussions from the Genetic Analysis Workshop 19

**DOI:** 10.1186/s12863-015-0317-6

**Published:** 2016-02-03

**Authors:** Arne Schillert, Stefan Konigorski

**Affiliations:** Institut für Medizinische Biometrie und Statistik, Universität zu Lübeck, Ratzeburger Allee 160, 23562 Lübeck, Germany; Molecular Epidemiology Research Group, Max Delbrück Center for Molecular Medicine in the Helmholtz Association, Robert-Rössle-Straße 10, 13125 Berlin-Buch, Germany

## Abstract

For Genetic Analysis Workshop 19, 2 extensive data sets were provided, including whole genome and whole exome sequence data, gene expression data, and longitudinal blood pressure outcomes, together with nongenetic covariates. These data sets gave researchers the chance to investigate different aspects of more complex relationships within the data, and the contributions in our working group focused on statistical methods for the joint analysis of multiple phenotypes, which is part of the research field of data integration. The analysis of data from different sources poses challenges to researchers but provides the opportunity to model the real-life situation more realistically.

Our 4 contributions all used the provided real data to identify genetic predictors for blood pressure. In the contributions, novel multivariate rare variant tests, copula models, structural equation models and a sparse matrix representation variable selection approach were applied. Each of these statistical models can be used to investigate specific hypothesized relationships, which are described together with their biological assumptions.

The results showed that all methods are ready for application on a genome-wide scale and can be used or extended to include multiple omics data sets. The results provide potentially interesting genetic targets for future investigation and replication. Furthermore, all contributions demonstrated that the analysis of complex data sets could benefit from modeling correlated phenotypes jointly as well as by adding further bioinformatics information.

## Introduction

For Genetic Analysis Workshop (GAW) 19, a large collection of different types of data were provided [1]. Researchers were able to use both systolic (SBP) and diastolic blood pressure (DBP) phenotypes, measured at multiple time points, gene expression measures, and sequencing data, as well as single nucleotide polymorphisms (SNPs) from families and unrelated individuals. This enabled participating researchers to investigate a multitude of complex questions, which often involved combining data from various sources.

**Fig. 1:**
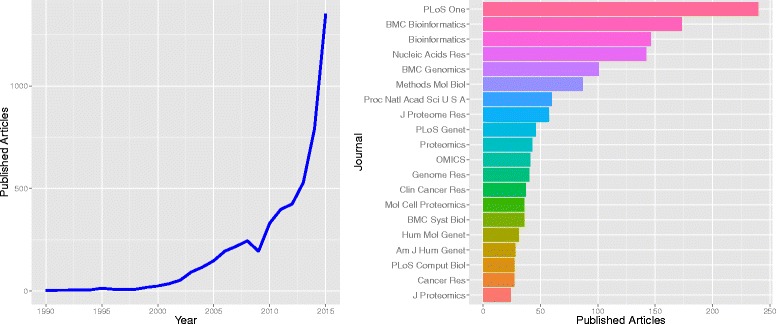
Results of PubMed literature search. Results of a literature search on PubMed on June 25, 2015, for articles published between January 1, 1990 and June 1, 2015, containing any of “(data integration OR joint model OR joint analysis OR multiple phenotypes OR multivariate model OR multivariate statistics)” as well as “(omics OR NGS OR high-throughput OR xx)”, where *xx* was any permutation of 2 omics measures from genomics, transcriptomics, epigenomics, proteomics, and metabolomics, each parameterized by different possible keywords. The number of retrieved articles in 2015 *(left panel)* is multiplied by 2.4 to predict published articles in 2015. The top 20 journals with the most publications are shown in the *right panel*

As a clarification at the beginning of this overview, *phenotype* is commonly used in the literature and the GAW19 contributions as a synonym of any measured nongenotypic variable, and we subscribe to this use in the following. The analysis of multiple phenotypes has been a recurring topic in past GAWs [2–4], and has caught more widespread interest in recent years as a result of technological advances that enable the collection of multiple phenotypes and multiple omics data at a larger scale. Recent reviews [5–7] provide an overview of statistical and computational methods for the integration of different omics data sets, for example, genomics, transcriptomics, epigenomics, proteomics, metabolomics, and phenomics. The methods are commonly referred to as *data integration* approaches, which aim at integrating the information of multiple levels of molecular measures—that is, multiple phenotypes—into 1 analysis. Possible ways to classify the existing approaches are to distinguish *multistaged* and *meta-dimensional approaches* [5], where multistaged analysis refers to a sequential analysis of associations between different data sources that are overlayed in a final analysis step. Meta-dimensional approaches, on the other hand, could be described by the attempt to build a joint model of all available data. In both approaches to data integration, efficient computational approaches to combine the large amount of data are necessary, and as a result, a large part of the methodological development stems from the bioinformatics community. This is illustrated by the results of a literature search for published articles describing specific forms of joint analysis of multiple phenotypes (Fig. 1). This *data integration* was addressed by the contributions in the GAW19 working group on the joint analysis of multiple phenotypes, but also aspects of the *analysis of dependent variables* were used. In this summary paper, we highlight the statistical methods presented in this working group, and discuss their contribution and value, in addition to the predominantly bioinformatics-driven perspective, on the analysis of multiple phenotypes.

## Blood pressure and gene expression as multiple phenotypes

**Fig. 2:**
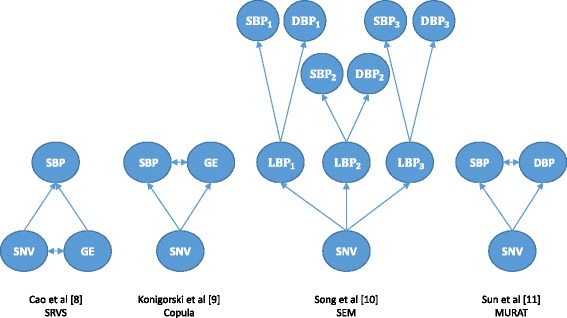
Simplified sketches of the underlying biological models and assumptions of the working group papers. *BP*_*i*_, BP measure at the *i* ^th^ visit; DBP, diastolic blood pressure; GE, gene expression; LBP, latent variable affecting both systolic and diastolic blood pressure; MURAT, multivariate rare-variant association test; SBP, systolic blood pressure; SEM, structural equation modeling; SNV, single nucleotide variant; SRVS, sparse representation-based variable selection. For a more detailed presentation of the models, please refer to the original articles

A first question for the analysis of multiple phenotypes can be: What are the multiple phenotypes that are investigated, and what is the motivation for a joint analysis? This can then be followed by more detailed questions as to how the multiple phenotypes are analyzed statistically, and whether, for example, they are considered as dependent outcome variables or as covariates on the same level as genotypes. Common to all contributions of this working group [8–11] was the search for functional single nucleotide variants (SNVs) influencing the blood pressure phenotypes, with different motivations and approaches for integrating multiple phenotypes into the analysis. SBP and DBP show a high correlation, with correlation coefficients between 0.5 and 0.8, depending on the adjustment applied for covariates. When searching for a common underlying genetic background, pleiotropic SNVs [12] influencing both blood pressure measurements, or different SNVs in the same gene, which are in high linkage disequilibrium and influencing either blood pressure (BP), can explain some of the dependence between SBP and DBP. In addition, the dependence between SBP and DBP might be partially explained by genetic effects, which are mediated by intermediate phenotypes such as, for example, gene expression. Hence, the available gene expression measures can be seen and were used as a further phenotype, which is also correlated with BP [13, 14], and the search for pleiotropic SNVs could target to identify variants influencing both BP and the gene expression of their gene (or another gene). Alternatively, gene expression level can be investigated as another predictor in addition to SNVs, to identify more complex biomarkers based on both SNVs and gene expression, and again to build more biologically meaningful models. SNVs and biomarkers identified from such analyses might be especially helpful to understand parts of the complex biological processes how genetic factors can affect BP. With BP being an important clinical and easily measurable variable with relevance for cardiovascular disease and other diseases, such as stroke or kidney damage, investigating longitudinal BP profiles and their underlying genetics is another relevant aspect of multiple phenotypes analysis, which was considered in the contributions. Figure 2 shows a simplified illustration of the underlying biological models of the working group papers.

Building on the above arguments, it is intuitive that a joint analysis of multiple BP and gene expression phenotypes together with the underlying genotypes may allow building a biologically more meaningful model. This can help to not only increase the face validity and confidence in findings from the analyses, but can also allow estimating the genetic effects more efficiently with higher power.

## Investigated phenotypes, transformations, and adjustments

All 4 contributions looked at BP as an end point. However, 1 group applied a log-transformation [11] to BP; 2 groups [8, 11] applied standard adjustments for the nongenetic covariates age, sex, and smoking; and the other 2 groups [9, 10] adjusted BP for the effect of antihypertensive medication and other nongenetic covariates using a censored regression model [15]. Furthermore, 1 group [10] looked at longitudinal effects, and 2 groups [8, 9] considered SBP only but included gene expression measures, which made a comparison of identified SNVs difficult. Table 1 provides a brief overview of the analyzed samples and data, and shows the employed statistical models, implementation, and main findings of all contributions.

**Table 1:** Overview of the analyzed sample and data in the contributions

Ref. #	Contribution	Sample	BP data	GE and genetic data	Method	Software	Main findings
[8]	Cao et al. [2015]	*n* = 397 individuals in 46 families, from family data set	Real data: SBP at time point 3	GE and SNP data: k = 11,522 transcripts, l = 354,893 SNPs	SRVS	Matlab-toolbox *SRVS*	Of top 1000 variables associated with BP, 575 are SNPs and 425 are GE, 302 have plausible relevance for BP, 173 are associated with body weight, and 84 associated with left ventricular contractility
[9]	Konigorski et al. [2015]	*n* = 81 unrelated individuals, from family data set	Real data: SBP at time point 1	GE and WGS data on chromosome 19: k = 848 transcripts, l = 68,727 SNVs	Copula	R functions, available upon request	Higher power of bivariate copula models compared to univariate regression and univariate SKAT, SKAT-O
							Identification of 5 SNVs in CEACAM5 gene relevant for SBP, and 1075 *cis*-eQTLs relevant for GE
[10]	Song et al. [2015]	*n* = 1389 individuals from family data set	Real data: SBP and DBP at time points 1–3	SNP data: l = 460,359 SNPs	SEM	R-package *strum*	The 2 tested models (autoregressive and latent growth curve) show similar ranking of relevant SNPs
							Identification of 10 SNPs related to both SBP and DBP, mostly on chromosome 1
[11]	Sun et al. [2015]	*n* = 1851 unrelated individuals, from unrelated data set	real data: SBP and DBP	WES data: l = 152,337 SNVs	MURAT	R functions, available upon request	Multivariate tests tend to give smaller *p* values than the univariate SKAT, and can improve power
							Identification of 2 SNPs in CYP4A22 and near APOC4, which were previously reported to be associated with BP

*BP* blood pressure, *eQTL* expression quantitative trait locus, *GE* gene expression, *MURAT* multivariate rare-variant association test, *SBP/DBP* systolic/diastolic blood pressure, *SEM* structural equation modeling, *SKAT* sequence kernel association test, *SKAT-O* optimal sequence kernel association test, *SNP* single nucleotide polymorphism, *SNV* single nucleotide variant, *SRVS* sparse representation variable selection, *WES* whole exome sequence, *WGS* whole genome sequence

## Dealing with the high dimensionality of the data

For genome-wide association studies (GWAS) in general, a paramount question is how to approach the high dimensionality of SNVs, with solutions of either testing SNVs or sets of SNVs sequentially, or by using some dimension reduction approach or penalized model to analyze all the data simultaneously. Whether a statistical method is applicable to high dimensional data, is dependent on whether it is computationally fast enough to fit a large number of models, sequentially testing each SNV, or whether it is both computationally fast enough and able to fit 1 very high-dimensional model including all SNVs (in the genome or in a chromosome, for example). This challenge is amplified when multiple phenotypes are considered, which increases the dimensionality problem further. Three of the contributions [9–11] approached this issue by restricting the analysis to prespecified models (see Fig. 2) with assumed relationships between phenotypes and SNVs. These models were then fitted sequentially for different SNVs or sets of SNVs with copula models [9], structural equation models (SEM) [10], or multivariate linear mixed effect regression [11]. As another novel approach to the high dimensionality, Cao et al. [8] used a sparse representation based variable selection (SRVS) to extract relevant genes based on signatures from the entire data, including all SNVs and all gene expressions. In this regard, all approaches could be described as different forms of meta-dimensional approaches, with either using a sparse statistical model and analyzing all the SNP and gene expression data simultaneously, or by posing hypothesis-based restrictions and testing triplets of SBP, DBP, and SNVs or of SBP, gene expression, and SNVs sequentially. In more detail, Cao et al. [8] analyzed 11,522 gene expression measures, 354,893 SNPs and 6 nongenetic variables simultaneously. Konigorski et al. [9] and Song et al. [10], each tested models incorporating multiple phenotypes (SBP and 1 gene expression measure [9], or 3 longitudinal SBP and 3 DBP measures [10]) together with 1 SNV/SNP, sequentially for 68,727 SNVs [9] and 460,359 SNPs [10]. Finally, Sun et al. [11] tested SBP and DBP conditional on the nongenetic covariates for the association with 152,337 SNVs sequentially, and with multimarker tests allocating these SNVs into 10,886 genes or 13,094 windows and testing them sequentially.

As another view on the approaches, Konigorski et al. [9], Song et al. [10], and Sun et al. [11] applied new methodologies and implementations for models of multiple dependent outcome measures to integrate BP and gene expression as phenotypes in the analysis. On the other hand, Cao et al. [8] looked at gene expression at the same level as genotypes to derive joint signatures relevant for BP, and applied a new approach to extend the literature on how to integrate and mine multiple covariates for interesting structures. Hence, the approaches focus on slightly different aspects of transcriptional and translational processes.

Additional hypothesis-based restrictions were sometimes used to decrease the number of tested hypotheses and the number of estimated parameters; for example, the assumption that the effects of the same variant on different phenotypes have the same correlation [11] or the restriction to analyze *cis*-acting SNVs with respect to gene expression and disregard *trans*-acting SNVs [9].

## Classical multiple phenotype analysis

A multivariate method in the classical sense was applied by Sun et al. [11]. Motivated by the search for pleiotropic genetic variants, which might help to shed more light on the genetic architecture of complex traits, they used their recently developed multivariate rare-variant association test (MURAT) to test for associations between BP and genetic variants. The underlying model of MURAT relates multiple phenotypes to a group of genotypes and covariates with a multivariate linear mixed effect model, assuming that the phenotypes are randomly distributed yet correlated and that effect of the genetic variants is normally distributed. Restrictions to the correlation structure involve the assumption that effects of different variants are uncorrelated but that the effect of the same variant on different phenotypes may be correlated. In the analysis, Sun et al. used SBP and DBP as highly correlated phenotypes to be associated with the sequencing data of unrelated individuals. They applied MURAT to the sequence data of the 1943 unrelated individuals and log-transformed BP measurements, which showed a correlation of *r* = 0.542. In their analysis, the authors compared the results with the frequently used sequence kernel association test (SKAT) [16]. Both tests were first used as single-variant tests, restricted to exomic variants on odd-numbered chromosomes with at least 4 carriers. For the application as region-based tests, they used 10,866 gene regions based on the hg19 annotations and also 13,094 nonoverlapping windows of 30 kb. Additionally, the authors looked at 15 candidate genes known to be associated with hypertension. A score type statistic with analytically computable *p* values allows the application in genome-wide analyses. Besides identifying potentially interesting candidate SNPs for hypertension, the authors found evidence for an increased power when applying the multivariate test, compared to the univariate SKAT method.

Song et al. [10] used a SEM method for pedigrees [17] allowing for measurement error of the observed BP values, to search for SNPs which affect the BP changes over time. In the measurement part of the model, SBP and DBP are assumed to be linearly related to the SNP and the latent variable, as illustrated in Fig. 2, assuming multivariate normality [17]. In the structural part of the model, the relationship between the 3 latent variables was modeled in 2 ways, with a first-order autoregressive model as well as a latent growth curve model. More specifically, in the first-order autoregressive model, the genotype affects only the first latent variable and all following variables depend only on their respective predecessor, and in the latent growth curve model, the effect of the SNP on the latent variables is constant. With a score test developed from their original R package *strum* [18], the authors tested the effect of each SNP separately on the BP traits. Because of a vast amount of missing data, Song et al. excluded the fourth time point from their analysis. In their analysis, the authors found weak associations signals for 10 SNPs in both models. The SNPs have not been reported in previous GWAS and could be investigated further in future studies. These results indicate that with the computationally efficient score test, SEMs of complex relationships between multiple phenotypes can be investigated with *strum* on a genome-wide scale.

## Data integration methods of multilevel biological data

Two of our groups incorporated data from different omics technologies in the form of genotypes and gene expression to make use of more information. Both used the provided family data set as it contained both genotype and gene expression data.

Konigorski et al. followed up on their work from GAW18 [15] and extended their analysis to gene expression data [9] for a more biologically meaningful model, and to the analysis of rare variants. In their model, the dependence between the gene expression of a transcript with BP is used for building joint models of both phenotypes conditional on genetic variants. The relationship between gene expression and BP is assumed to be undirected, because it is not clear a priori whether for a particular transcript, the effect is from gene expression on BP or vice versa. For example, the mRNA expression of some genes can be expected to influence BP levels through complex translational processes, but also, BP could be thought of as an indicator of, for example, the stress level of an individual, which can be characterized by changes in the blood level of different hormones and proteins. These hormone levels could in turn interact with mRNA transcripts and modulate gene expression, resulting in an “effect” of BP on the gene expression levels. The authors used copula functions to model the joint distribution of SBP and gene expression conditional on SNVs, separately for all SNVs within the gene boundaries, to investigate genetic effects on both phenotypes while considering their dependence structure. In the marginal models, the SNV is linearly related to the phenotype. They restricted their analysis to chromosome 19, as it contains the transcript/gene with the highest association with SBP and hence a joint model could benefit the most compared to a univariate model. Genetic variants had to have at least 3 copies and must be located within 5 kb of a gene to be considered. Hence, for the effect on gene expression, only *cis*-acting effects were considered. They compared their results with results from single-marker univariate models of SBP and gene expression and also the gene-based SKAT [16] and SKAT-O (optimal sequence kernel association test) [19]. While there was no indication for inflated type 1 errors under the proposed copula approach, the results indicate that joint models using copula functions can estimate the genetic effects of both common and rare variants more efficiently and with higher power compared to standard univariate regression models as well as the popular multimarker tests of SKAT and SKAT-O.

Cao et al. applied their previously developed SRVS [20] to identify signatures from SNPs and mRNA expression which are associated with BP [8]. After regressing out the nongenetic covariates from the observed BP, the resulting residuals are related to all SNPs and transcripts in a linear regression model. This high-dimensional model is solved with their SRVS algorithm to select SNPs or transcripts. In the approach, combinations of SNPs and transcripts are repeatedly randomly selected and used for a least-squares optimization for minimal regression coefficients. Variables with nonzero regression coefficients are considered as selected. For the top 1000 selected variables, they performed a bioinformatics analysis to investigate the potential biological relevance of these variables. For each selected SNP or gene they looked for associations with BP (logarithm of odds [LOD] score >3) using the rat genome database tool [21] based on the Human Genome Assembly GRCh37. Approximately 56 % of the SNPs were identified as BP related and could be interesting targets for follow up in future studies. These results show the applicability of the SRVS algorithm to a large data set including multiple data sources with the potential to identify interesting biomarkers.

## Discussion

When summarizing insights from the 4 contributions, it was demonstrated that substantially different joint statistical models of multiple phenotypes are available for analysis, that their implementation is computationally efficient enough for an analysis on a genome-wide scale, and can be applied to multiple omics data sets without modification [8] or by posing further hypothesis-based constraints or through extensions of the approaches [9–11]. Complex molecular relationships of interest can be hypothesized from previous knowledge and investigated, or also, the entire data can be explored as a starting point to deriving meaningful signatures relevant for BP. All approaches analyzed the real data with the focus on applying new methodologies, showing their potential usefulness, and comparing the results to established approaches. In all comparisons to standard univariate approaches, promising results were described which suggest that using the information from multiple phenotypes can increase the power for identifying relevant genetic variants, even when considering a power decrease in joint models because of a smaller sample size with complete information on all phenotypes. For example, when using gene expression data from unrelated individuals of the San Antonio family study, the sample size available for analysis was *n* = 81 [9]. Future work in this area could investigate adapted imputation models and extend the approaches to model the family structure, to increase the available sample size for analysis. Because the simulated phenotypes weren’t used in the analyses, future studies are also needed to evaluate the empirical type 1 error and power of the proposed test statistics.

As Cao et al. [8] demonstrated, subsequent bioinformatics analysis can help in the interpretation of the obtained results, and complement both model-based statistical approaches and unsupervised methods. Starting from identified associations of genetic variants, using causal models or developing appropriate goodness-of-fit tests for effects in different directions could each be very interesting roads for future extensions and future work for the fine-mapping of effects in the complex biological systems.

Finally, in addition to the methodological development of multivariate statistical approaches, an inherent premise in all contributions was that a careful selection, adjustment, and statistical modeling of each of the multiple phenotypes is essential for obtaining reliable results. With this, what can be more intuitive than to recall the classical genetic definition of a phenotype, and model the multiple observed traits of a phenotype jointly, which each contribute and explain parts of the entire organism.
